# Contributions of Embryonic HSC-Independent Hematopoiesis to Organogenesis and the Adult Hematopoietic System

**DOI:** 10.3389/fcell.2021.631699

**Published:** 2021-02-18

**Authors:** Wen Hao Neo, Michael Lie-A-Ling, Muhammad Zaki Hidayatullah Fadlullah, Georges Lacaud

**Affiliations:** Stem Cell Biology Group, Cancer Research UK Manchester Institute, The University of Manchester, Macclesfield, United Kingdom

**Keywords:** yolk sac, lineage tracing, embryonic hematopoiesis, organogenesis, macrophage, hematopoietic waves, HSC-independent hematopoiesis

## Abstract

During ontogeny, the establishment of the hematopoietic system takes place in several phases, separated both in time and location. The process is initiated extra-embryonically in the yolk sac (YS) and concludes in the main arteries of the embryo with the formation of hematopoietic stem cells (HSC). Initially, it was thought that HSC-independent hematopoietic YS cells were transient, and only required to bridge the gap to HSC activity. However, in recent years it has become clear that these cells also contribute to embryonic organogenesis, including the emergence of HSCs. Furthermore, some of these early HSC-independent YS cells persist into adulthood as distinct hematopoietic populations. These previously unrecognized abilities of embryonic HSC-independent hematopoietic cells constitute a new field of interest. Here, we aim to provide a succinct overview of the current knowledge regarding the contribution of YS-derived hematopoietic cells to the development of the embryo and the adult hematopoietic system.

## Introduction

**FIGURE 1:**
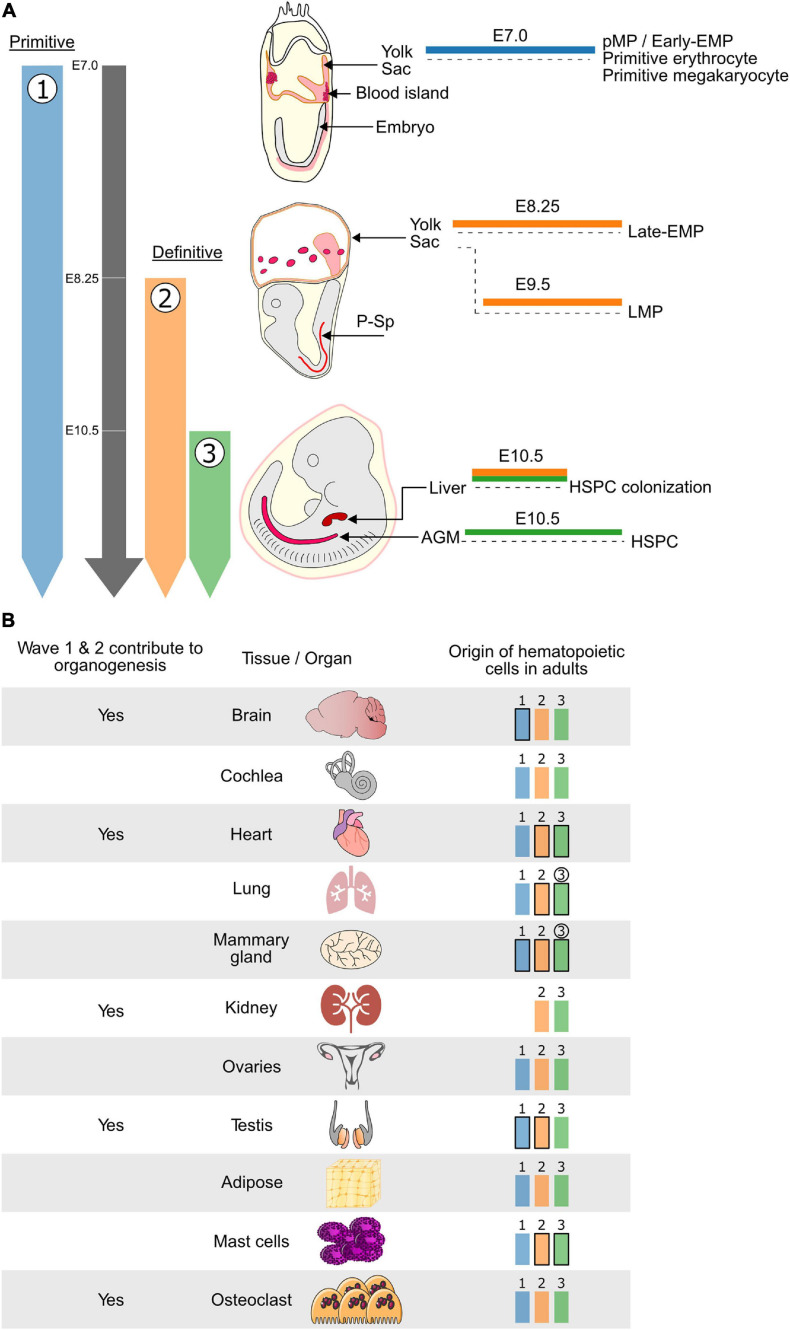
The hematopoietic system is established in developmental waves that differentially contribute to embryonic organogenesis and the adult hematopoietic system. **(A)** Schematic representing the timing and embryonic sites of hematopoietic activity during mouse development. The three waves of hematopoiesis are represented in Blue (wave 1: primitive erythrocytes, megakaryocytes, macrophages and Early EMP), Orange (wave 2: Late EMP and LMP) and Green (wave 3: HSPC). Wave 1 is known as the primitive wave while waves 2 and 3 constitute the definitive waves of hematopoiesis. Waves 1 and 2 which do not generate HSCs originate in the Yolk Sac while wave 3, which generates the HSPCs, is initiated in the AGM region of the embryo proper. Cells from both waves 2 and 3 can colonize the fetal liver where they can mature and expand before moving to their final destination. **(B)** (Left) HSC-independent macrophages which originate from the first two hematopoietic waves have been shown to play important roles during embryonic development of several tissues and organs. (Right) In addition to the wave 3 (Green) HSC-derived hematopoietic cells, the HSC-independent hematopoietic cells generated during wave 1 (Blue) and 2 (Orange) persist, to varying degrees, in adult tissues and organs. Waves that contribute most of the hematopoietic cells are depicted in bold and waves for which the contribution increases overtime are circled. Blue: hematopoietic wave 1 (primitive), Orange: hematopoietic wave 2 (Late-EMP/LMP), Green: hematopoietic wave 3 (HSC). E, embryonic day; Mφ, macrophage; HSC, hematopoietic stem cell; EMP, erythroid–myeloid progenitor; LMP, lymphoid–myeloid progenitor; HSPC, hematopoietic stem and progenitor cell; P-Sp, para-aortic splanchnopleura; AGM, aorta-gonad-mesonephros.

In mammals, the hematopoietic system is established during embryogenesis in three consecutive overlapping waves (Dzierzak and Bigas, 2018). In mice, the first wave, also termed primitive hematopoiesis, is initiated around embryonic day (E)7 and produces unipotent precursors that give rise to either primitive-erythrocytes, -megakaryocytes, or -macrophages (Palis et al., 1999; Tober et al., 2007). The primitive macrophage precursors have also been named primitive myeloid precursors (pMP). Furthermore, it has also been reported that the first wave may give rise to c-Myb independent erythroid–myeloid progenitors (early EMP) (Hoeffel et al., 2015). However, so far, macrophage (microglia) but not erythrocyte potential has been experimental confirmed for these so-called early EMP (Wittamer and Bertrand, 2020). Henceforth, we will refer to wave 1 myeloid cells as pMP/early EMP. The second wave of hematopoiesis marks the onset of definitive (erythroid) hematopoiesis and sees the emergence of both EMPs around E8.25 (late-EMPs) (McGrath et al., 2015; Palis, 2016), and lymphoid–myeloid progenitors (LMPs) (Adolfsson et al., 2005; Boiers et al., 2013) around E9.5. Around E10.5 the third wave generates both hematopoietic stem and progenitor cells (HSPC) (Figure 1A). The hematopoietic stem cells (HSCs) subsequently play a central role in maintaining the hematopoietic system for the lifetime of the organism (Medvinsky et al., 1993; Muller et al., 1994). Hematopoietic cells of all waves are generated from the mesoderm, which is known to give rise to both endothelial and hematopoietic lineages (Davidson and Zon, 2000; Dzierzak and Bigas, 2018). For the definitive hematopoietic waves (waves 2 and 3), it is now well established that hematopoiesis occurs via an endothelial-to-hematopoietic transition (EHT) from a specialized endothelial subpopulation known as hemogenic endothelium (HE) (Jaffredo et al., 1998; Zovein et al., 2008; Chen et al., 2009; Eilken et al., 2009; Lancrin et al., 2009; Boisset et al., 2010; Lacaud and Kouskoff, 2017; Garcia-Alegria et al., 2018; Ottersbach, 2019). The cellular origin of the first wave of primitive hematopoiesis is still disputed. It is unclear whether primitive hematopoiesis emerges directly from mesoderm, a hemangioblast, a HE, or another type of precursor (Amaya, 2013; Myers and Krieg, 2013). However, several recent studies suggest that primitive hematopoiesis (wave 1) is generated through a HE(-like) intermediate that has been termed hemogenic angioblast (Lancrin et al., 2009; Stefanska et al., 2017; Garcia-Alegria et al., 2018). Despite this potential common cellular origin, not all waves originate from the same anatomical site. The first two waves arise extra embryonically in the yolk sac (YS). In contrast, the third wave mainly takes place in the dorsal aorta within the aorta-gonad-mesonephros (AGM) region of the embryo, where HSC arise within so-called intra-aortic hematopoietic clusters (IAHC) (Boisset et al., 2010; Dzierzak and Bigas, 2018; Ottersbach, 2019). The HSC mature and amplify in the fetal liver (FL) before taking up residence in the bone marrow (BM).

Initially, the YS waves of hematopoiesis were thought to provide an essential but transient blood supply for the embryo destined to be replaced by HSC-derived cells. However, there is accumulating evidence that HSC-independent blood cells can make significant contributions to the adult hematopoietic system. Furthermore, it is also becoming evident that in the embryo, the HSC-independent hematopoietic waves can play essential roles beyond erythrocyte-mediated oxygen exchange and early immune surveillance. These roles have been mainly assigned to myeloid-derived populations that have been shown to play crucial roles in embryonic organogenesis.

Here we provide a concise overview of the recent findings obtained in mouse models regarding the contribution of HSC-independent hematopoiesis to embryonic organogenesis and the adult hematopoietic system.

## Resolving the Role and Origin of Hematopoietic Populations

The overlapping and transient nature of the different hematopoietic waves makes it challenging to determine their individual contributions towards organogenesis and the adult hematopoietic system. Transgenic mouse knockout (KO) and fate mapping models have been instrumental in shaping our current understanding of the contribution of the different hematopoietic waves towards the hematopoietic system (Table 1). KO models (via gene deletion/mutation or lineage-specific activation of diphtheria toxin) provide functional information for specific populations, while lineage tracing models (typically using fluorescent proteins) highlight the contribution of specific populations. However, the perfect model to delineate the hematopoietic waves does not exist, and it is important to consider the target cell type, wave specificity and labeling efficiency of the models used when interpreting results.

**TABLE 1:** Mouse model for studying the contribution of different hematopoietic waves to hematopoietic system.

Knockout models	Function	Affected wave	Major impact on hematopoietic waves	References
*Csf1r* (Dai et al., 2002)	Early/Late-EMP differentiation	1, 2	Lack of early EMP and reduced late-EMP contribution to TRM	Ginhoux et al., 2010; Hoeffel et al., 2012
*Csf1* (null mutation) (Marks and Lane, 1976)	Early EMP differentiation	1	Reduced early EMP contribution to TRM	Cecchini et al., 1994; Kondo and Duncan, 2009; Easley-Neal et al., 2019
*Myb* (Mucenski et al., 1991)	Late-EMP and HSC differentiation	2, 3	Lack of late-EMP and HSC contribution to TRM	Schulz et al., 2012; Hoeffel et al., 2015
*Nur77* (Lee et al., 1995)	BM monocytes differentiation		Reduced BM monocytes/macrophages contribution to TRM	Hanna et al., 2011
*Plvap* (Rantakari et al., 2015)	FL macrophage migration		Reduced FL monocytes/macrophages contribution to TRM	Rantakari et al., 2016
*Ccr2* (Hanna et al., 2011)	BM monocytes/macrophages migration		Reduced BM monocytes/macrophages contribution to TRM	Boring et al., 1997
*KitL* (Ding et al., 2012)	Late-EMP and HSC maintenance	2, 3	Reduced late-EMP and HSC contribution to TRM	Ajami et al., 2007; Azzoni et al., 2018
*Cx3cr1* (Jung et al., 2000)	Mediates monocyte retention in the BM		None	Imai et al., 1997; Jacquelin et al., 2013
*Pu.1* (Scott et al., 1994; McKercher et al., 1996)	YS myeloid differentiation and HSC maintenance	1, 2, 3	Lack of EMP and HSC contribution to TRM	Scott et al., 1994; Olson et al., 1995; McKercher et al., 1996; Kim et al., 2004; Iwasaki et al., 2005; Kierdorf et al., 2013
*Runx1* (Okuda et al., 1996; Wang et al., 1996a; North et al., 1999)	Master regulator of hematopoiesis, expressed from HE onwards	1, 2, 3	Lack of EMP and HSC	Okuda et al., 1996; Wang et al., 1996a; North et al., 1999
*Cbf*β (Sasaki et al., 1996; Wang et al., 1996b; Niki et al., 1997)	Essential RUNX1 co-factor	1, 2, 3	Lack of EMP and HSC	Sasaki et al., 1996; Wang et al., 1996b; Niki et al., 1997
*Cbf*β (*Tie2-Cbf*β) (Miller et al., 2002)	Essential RUNX1 co-factor	1, 2, 3	Lack of HSC	Chen et al., 2011
*Cbf*β (*Ly6a-Cbf*β) (Chen et al., 2011)			Lack of EMP	Chen et al., 2011

**Constitutive fate mapping models**	**Relevance**	**Labeling efficiency (assay time point)**				**References**
		**LMP (Wave 2)**	**pMP/Early EMP (Wave 1)**	**Late-EMP (Wave 2)**	**HSC (Wave 3)**	

*S100a4* (Bhowmick et al., 2004) (Transgene)	Active in FL monocytes and not in FL macrophages	Not done	∼20% (5wo)	64.5 ± 6.7% (5wo)	Not done	Hoeffel et al., 2015
		Not done	∼20% (Adult)	∼60% (Adult)	∼100% (Adult)	Hashimoto et al., 2013
*Flt3* (Benz et al., 2008) (KI)	Active in HSC progeny	Not done	Not done	Not done	∼80% (NB)	Hoeffel et al., 2015
		Not done	Not done	∼20% (Adult)	∼80% (Adult)	Hashimoto et al., 2013
		Not done	<2% (P8 onwards)	10–30% (P8 onwards)	∼80% (P8 onwards)	Gomez Perdiguero et al., 2015
		Not done	Not done	10–20% (4wo)	∼80% (4wo)	Schulz et al., 2012
*Ms4a3* (Liu et al., 2019) (KI)	Active in GMP	Not done	None (NB onwards)	Trace (NB onwards)	BM GMP: 68.7 ± 1.58% (Adult)	Liu et al., 2019
*Tnfrs11a* (Maeda et al., 2012) (KI)	Essential for osteoclast development. Tracks YS progenitors.	Not done	∼80% (E14.5)	∼90% (6wo)	∼10% (E14.5 onwards)	Mass et al., 2016
*Tnfrs11a* (Percin et al., 2018) (KI)		Not done	Not done	∼90% (Adult)	None (Adult)	Percin et al., 2018
*Rag1* (McCormack et al., 2003) (KI)	Lymphoid-specific activity	∼100% (E14.5)	Trace (E14.5)	<5% (E14.5)	Not done	Boiers et al., 2013

**Inducible fate mapping models**	**Relevance**	**Pulse timepoint**	**Labeling efficiency (assay time point)**			**References**
			**pMP/Early EMP (Wave 1)**	**Late-EMP (Wave 2)**	**HSC (Wave 3)**	

*Csf1r* (Qian et al., 2011) (Transgene)	Mainly a myeloid marker	E8.5	63.2 ± 5.6% (E13.5)	Trace (E13.5)	Not done	Hoeffel et al., 2015
			∼60% (E11.5)	Marked (E12.5)	Not done	Gomez Perdiguero et al., 2015
*Cx3cr1* (Yona et al., 2013) (KI)	Mainly a myeloid marker	E9.0	∼40% (E16.0); ∼30% (NB onwards)	Trace (6wo onwards)	Trace (6wo)	Hagemeyer et al., 2016
*Runx1* (Samokhvalov et al., 2007) (KI; driven by P2/Runx1b promoter)	Master regulator of hematopoiesis, expressed from HE onwards	E7.25-E7.5	∼30% (E10.5)	<5% (8wo)	<3% (8wo)	Ginhoux et al., 2010
			Not done	Not done	none (9–12mo)	Samokhvalov et al., 2007
			∼30% (E10.5); ∼20% (E13.5)	Not done	Not done	Hoeffel et al., 2012
		E7.5	22.2 ± 0.9% (E13.5)	< 5% (E13.5); <10% (E16.5)	Trace (E13.5)	Hoeffel et al., 2015
			Not done	∼10% (8wo)	∼10% (8wo)	Ginhoux et al., 2010
			Not done	Not done	1–10% (9–12mo)	Samokhvalov et al., 2007
			Not done	∼12.5% (8wo)	∼7.5% (8wo)	Hoeffel et al., 2012
		E8.5	∼15% (E13.5); <5% (E16.5)	∼25% (E13.5); ∼30% (E16.5)	1–3% (E11.5); <5% (E13.5)	Hoeffel et al., 2015
			Not done	∼30% (8wo)	∼30% (8wo)	Ginhoux et al., 2010
			Not done	Not done	1–50% (9–12mo)	Samokhvalov et al., 2007
			Not done	∼30% (8wo)	∼30% (8wo)	Hoeffel et al., 2012
		E9.5	<5% (E13.5)	∼15% (E13.5)	∼30% (E13.5)	Hoeffel et al., 2015
			Not done	∼20% (8wo)	∼30% (8wo)	Ginhoux et al., 2010
			Not done	Not done	50–100% (9–12mo)	Samokhvalov et al., 2007
			Not done	∼25% (8wo)	∼30% (8wo)	Hoeffel et al., 2012
*Tie2* (Gomez Perdiguero et al., 2015) (KI)	Endothelial marker (including HE) also expressed in subset of HSC and myeloid cells	E7.5	∼60% (E12.5)	∼40% (6–8wo)	∼40% (E12.5)	Gomez Perdiguero et al., 2015
		E8.5	∼30% (E12.5)	∼20% (6–8wo)	∼75% (E12.5)	
		E9.5	trace (E12.5)	∼10% (6–8wo)	∼80% (E12.5)	
		E10.5	none (E12.5)	∼5% (6–8wo)	∼40% (E12.5)	
*Kit* (Sheng et al., 2015) (KI)	Express in early HSPC and YS HE cells	E7.5	∼70% (E13.5)	Trace (E13.5)	Trace (6wo)	Sheng et al., 2015
		E8.5	∼70% (E13.5)	∼40% (E13.5)	∼60% (6wo)	
		E9.5	∼50% (6wo)	∼50% (6wo)	∼40% (6wo)	
*Cdh5* (Sorensen et al., 2009) (Transgene)	Endothelial marker (including HE)	E7.5	∼80% (E10.5); ∼90% (E14.5)	∼80% (E10.5)	<10% (E14.5)	Gentek et al., 2018a
		E10.5	Trace (E14.5)	Not done	∼80% (E14.5)	

*EMP, erythroid–myeloid progenitors; HSC, hematopoietic stem cells; TRM, tissue-resident macrophages; FL, fetal liver; BM, bone marrow; pMP, primitive myeloid precursors; HSPC, hematopoietic stem and progenitors; YS, yolk sac; LMP, lymphomyeloid progenitors; GMP, granulocyte-macrophage progenitors; HE, hemogenic endothelium; KI, knock-in; NB, new born; wo, week old; mo, month old.*

Knockout mouse models have demonstrated specific dependencies of (wave-specific) hematopoietic populations on distinct transcription factors and signaling pathways. *Csf1r* (Colony Stimulating Factor 1 Receptor, cytokine receptor) KO mainly disrupts the early EMP differentiation (wave 1) and to a lesser extent the late-EMP differentiation (wave 2) (Dai et al., 2002). Although these mice are viable, they display drastically reduced levels of microglia and YS macrophages (Ginhoux et al., 2010; Hoeffel et al., 2012). *Csf1* (Marks and Lane, 1976) (Colony Stimulating Factor 1, cytokine) null mice display a similar but milder phenotype, with varying degrees of microglia and YS macrophages depletion, due to partial compensatory effects of the alternative CSF1R ligand IL-34 (Wiktor-Jedrzejczak et al., 1990; Cecchini et al., 1994; Kondo and Duncan, 2009; Greter et al., 2012; Wang et al., 2012; Easley-Neal et al., 2019). *Myb* (Mucenski et al., 1991) (MYB Proto-Oncogene, transcription factor) KO disrupts late-EMP and HSC differentiation (wave 2 and wave 3) and results in anemia and embryonic lethality around E15.5 (Schulz et al., 2012; Hoeffel et al., 2015). A similar phenotype is observed in KitL (KIT Ligand, cytokine) KO mice which die perinatally (Ding et al., 2012). *Nur77* (Lee et al., 1995) (Nuclear Receptor Subfamily 4 Group A Member 1, nuclear receptor) KO is viable but lacks circulating monocytes due to disrupted BM HSC differentiation (Hanna et al., 2011). Disrupting cell migration has also emerged as a useful strategy. *Plvap* (Rantakari et al., 2015) (Plasmalemma Vesicle Associated Protein, membrane protein) and *CCR2* (Boring et al., 1997) (C-C Motif Chemokine Receptor 2, chemokine receptor) KOs are viable but respectively show impaired FL and BM monocyte migration (Rantakari et al., 2016). *Cx3cr1* (Jung et al., 2000) (C-X3-C Motif Chemokine Receptor 1, chemokine receptor) KO is viable but shows impairment of leukocyte migration (Imai et al., 1997; Jacquelin et al., 2013) regardless of their wave of origin. There are several other models which disrupt all waves of hematopoietic development. *Pu.1* (Scott et al., 1994; McKercher et al., 1996) (Spi-1 Proto-Oncogene, transcription factor) KO mice have defective YS myelopoiesis and HSC maintenance and die shortly after birth (Olson et al., 1995; Kim et al., 2004; Kierdorf et al., 2013). Deletion of *Runx1* (Okuda et al., 1996; Wang et al., 1996a; North et al., 1999) (RUNX Family Transcription Factor 1, transcription factor) or its essential co-factor *Cbf*β (Sasaki et al., 1996; Wang et al., 1996b; Niki et al., 1997) (Core-Binding Factor Subunit Beta, transcription factor) is embryonically lethal and results in the complete absence of hematopoiesis apart from primitive erythroid cells. These KOs are useful when coupled with targeted approaches. For example, the *Cbf*β KO model can be used to deplete EMP or HSC by combination with respectively *Ly6a-Cbf*β or *Tie2-Cbf*β rescue alleles (Chen et al., 2011).

Constitutive lineage tracing models rely on lineage-specific promoter activity to drive *Cre* recombinase expression, which in turn irreversibly activates or deletes a target gene (Hoess and Abremski, 1984; Sauer and Henderson, 1988). Such models have been established to trace long term lineage contribution of HSC- and YS-derived hematopoietic cells. *Flt3*-Cre, *Ms4a3*-Cre, *S100a4*-Cre predominantly track HSC progeny, albeit with several restrictions. *Flt3*-Cre (Schulz et al., 2012; Hashimoto et al., 2013; Gomez Perdiguero et al., 2015; Hoeffel et al., 2015) and *S100a4*-Cre (Hashimoto et al., 2013; Hoeffel et al., 2015) mark the majority of HSC-derived cells (>80%). However, both also mark some YS-derived hematopoietic cells (Table 1). In contrast, *Ms4a3*-Cre does not mark any YS cells from the first or second wave and only marks HSC-derived GMPs (∼70%) (Liu et al., 2019). The *Tnfrs11a*-Cre model is currently best suited to track YS hematopoiesis with little (Maeda et al., 2012; Mass et al., 2016) or no (Percin et al., 2018) HSC labeling. However, this model cannot distinguish the two YS waves of hematopoiesis. Currently, the only option to track LMP progeny is the *Rag1*-Cre fate mapping model (Boiers et al., 2013) which marks all FL B and T cells alongside a small number of myeloid cells.

Inducible tracing models [tamoxifen-inducible Cre-mediated recombination (Metzger et al., 1995; Feil et al., 1997)] add an extra layer of specificity that can overcome certain limitations of the constitutive models. This approach allows not only for reporter activation or gene deletion in specific cell types but also during a defined developmental time window. The latter has allowed for the specific marking of the first hematopoietic wave in the YS (pMP/early EMP) using multiple models (Table 1). In this context, a caveat of the *Csf1r*-Mer-iCre-Mer and *Cx3cr1*-CreER based systems is that they only label myeloid progeny (Gomez Perdiguero et al., 2015; Hoeffel et al., 2015; Hagemeyer et al., 2016). In contrast, *Tie2*-Mer-iCre-Mer, *Kit*-Mer-Cre-Mer, *Runx1*-Mer-Cre-Mer, and *Cdh5*-CreERT2 provide less restricted marking. Distinguishing progeny from late-EMP (wave 2) and HSC (wave 3) is still challenging, as illustrated in Table 1 (Samokhvalov et al., 2007; Ginhoux et al., 2010; Hoeffel et al., 2012, 2015; Gentek et al., 2018a).

## HSC-Independent Hematopoietic Cells Contribute to the Adult Hematopoietic System

In the adult hematopoietic system, several hematopoietic populations have been shown to consist of cells with an HSC-independent embryonic origin (Figure 1B). This has been best studied for tissue-resident macrophages (TRM), which were traditionally thought to be continuously replenished by BM HSC-derived monocytes. However, this view was challenged by the discovery of radiation-resistant and self-repopulating Langerhans cells (Merad et al., 2002), microglia (Ajami et al., 2007; Ginhoux et al., 2010), and alveolar macrophages (Guilliams et al., 2013; Hashimoto et al., 2013; Jakubzick et al., 2013) in BM transplantation and parabiosis studies. Subsequent lineage tracing studies have convincingly demonstrated that microglia are the progeny of myeloid cells produced during the first wave of hematopoiesis. *Csf1r*-Mer-iCre-Mer, *Cx3cr1*-CreER, *Runx1*-Mer-Cre-Mer, and *Kit*-Mer-Cre-Mer lineage tracing models all support the pMP/early EMP origin of microglia (Gomez Perdiguero et al., 2015; Hoeffel et al., 2015; Sheng et al., 2015; Hagemeyer et al., 2016). This has been further strengthened by the analysis of *KitL* and *c-Myb* KO models in which the number of late-EMP and HSC (waves 2 and 3) is drastically decreased, while the microglia population remains unaffected (Ding et al., 2012; Schulz et al., 2012; Azzoni et al., 2018).

It is now widely accepted that TRM populations do not have a unified common origin. Some tissues retain and maintain YS-derived cells while in others they are replaced or co-exist with BM-HSC-derived cells (for review Ginhoux and Guilliams, 2016; Mass, 2018; Wittamer and Bertrand, 2020). Below, we highlight recent findings concerning the persistence of YS-derived embryonic hematopoietic cells in adults.

### HSC-Independent Origin of Adult TRM

Alveolar macrophages (AM) and interstitial macrophages (IM) are two major subsets of lung TRM (Lehnert et al., 1985; Liegeois et al., 2018). Around E10.5, YS pMPs/early EMPs (Tan and Krasnow, 2016) seed in the primordial lung buds. Parabiosis, adoptive transfer, and lineage tracing experiments have shown that a subset of pMP/early EMP-derived IM (Hoeffel et al., 2012, 2015; Guilliams et al., 2013; Gomez Perdiguero et al., 2015; Tan and Krasnow, 2016; Liu et al., 2019) and AM (van de Laar et al., 2016; Li et al., 2020) persist into adulthood. The functional significance of these sub-populations, if any, is currently unknown. However, it has been shown that FL monocyte-derived TRM possess enhanced mitochondrial respiratory and glycolytic capacity (Li et al., 2020) versus their HSC-independent counterparts.

Adipose tissue macrophages (ATM), are the most abundant immune cells in adipose tissues and play a prominent role in regulating inflammation and insulin sensitivity (Russo and Lumeng, 2018). ATM, which can proliferate and self-renew, are partially derived from embryonic HSC-independent cells (E9.0 pulsed *Cx3Cr1-*CreER) (Hassnain Waqas et al., 2017; Waqas et al., 2017). Further studies using the *Ms4a3*-Cre fate-mapping model, which traces BM-HSC monocyte-derived cells (Jaitin et al., 2019; Liu et al., 2019), demonstrated that half of the white adipose tissue ATM is Ms4a3 positive under normal physiological conditions. Interestingly, a high-fat diet increases the proportion of HSC-derived monocytes within ATM population (Jaitin et al., 2019).

The *Ms4a3*-Cre fate-mapping model has also revealed that adult renal macrophages (RM) are of mixed origin (Schulz et al., 2012; Epelman et al., 2014; Gomez Perdiguero et al., 2015; Hagemeyer et al., 2016; Mass et al., 2016; Liu et al., 2019; Munro et al., 2019). Furthermore, YS-derived RM (E9.5 pulsed *Cx3cr1-*CreER) are more proliferative than their HSC-derived counterpart and their contribution to the kidney TRM population expands with age (Ide et al., 2020).

Osteoclasts are multinucleated myeloid cells that resorb bone tissue and are critical for the development, repair, and remodeling of the skeleton (Udagawa et al., 1990; Takahashi et al., 1994). The HSC-independent origin of osteoclast was first suggested by *ex vivo* co-culture experiments (Thesingh, 1986) and has been recently confirmed *in vivo* using *Csf1r-*Mer-iCre-Mer and *Cx3cr1-*CreER models (Jacome-Galarza et al., 2019; Yahara et al., 2020). Early/late EMP-derived osteoclasts not only persist but also expand in the adult BM (Yahara et al., 2020). Furthermore, a subpopulation of early/late EMP-derived osteoclasts was found to reside in the spleen. Interestingly, this spleen population can fuse with BM-derived monocytes creating long-lived osteoclast syncytia with a mixed origin (Jacome-Galarza et al., 2019; Yahara et al., 2020).

Based on CD206 and MHCII expression, three testis subpopulations of TRM can be distinguished. These populations possess different levels of phagocytic capacity (Lokka et al., 2020). Tracing (E8.5 pulsed *Csf1r*-Mer-iCre-Mer, E13.5 pulsed *Cx3cr1*-CreER) and KO (*Ccr2*, *Nur77*, *Plvap*) studies have shown that all three waves of hematopoiesis contribute to adult testis TRM. Strikingly, antibody-based macrophage depletion experiments have demonstrated that adult BM-derived cells play no part in testis TRM maintenance (Lokka et al., 2020).

Finally characterization of *Csf1* KOs, the *Csf1r-*EGFP constitutive tracing model, and E8.5 pulse-labeled *Csf1r-*Mer-iCre-Mer mice point to an HSC-independent origin for part of the TRM in adult cochlea (Kishimoto et al., 2019), ovaries (Jokela et al., 2020), and mammary glands (Gouon-Evans et al., 2000; Jappinen et al., 2019; Stewart et al., 2019).

### Beyond Tissue Resident-Macrophages: Mast- and Lymphoid-Cells

Mast cells (MC) can be classified into two groups. Connective tissue MC (CTMC) populate the skin, tongue, trachea, esophagus, adipose tissues, and peritoneal- and pleural cavities while mucosal MC (MMC) are found in the gut and respiratory mucosa. The exclusive BM HSC-derived (van Furth and Cohn, 1968) origin of MC was first challenged by transplantation assays that showed BM only minimally contributes to MC repopulation in MC-depleted hosts (Kitamura et al., 1977, 1978). Subsequent fate-mapping studies using *Csf1r*-Mer-iCre-Mer and *Runx1*-Mer-Cre-Mer suggested that the majority of MMC are derived from HSC, whereas CTMC are largely derived from the HSC-independent EMP (Li Z. et al., 2018). Interestingly, EMP-derived and HSC-derived MC have distinct transcriptional profiles suggesting distinct biological functions (Gentek et al., 2018a; Li Z. et al., 2018). If the CTMCs derived from HSC-independent cells can persist in significant numbers in the adult is unclear. *Csf1r*-Mer-iCre-Mer and *Runx1*-Mer-Cre-Mer fate-mapping studies suggest that they can, while *Cdh5*-CreERT2 based lineage tracing suggests a mostly fetal HSC origin of adult CTMC (Gentek et al., 2018a). These contradictory findings highlight that data from fate-mapping models should be interpreted with caution and that currently, no model can definitively distinguish the progeny of late-EMP from fetal HSC.

Finally, YS-derived lymphoid cells have also been found to persist into adulthood. Early B and T-cells [B1a (Yoshimoto et al., 2011; Kobayashi et al., 2014) and γδ T (Boiers et al., 2013; Gentek et al., 2018b) cells], a primary source of innate immunity in early embryo development (Yoshimoto et al., 2012), persist into adulthood and remain functionally distinct from their HSC-derived counterparts. The ontogeny and contribution of these YS-derived lymphocytes has been reviewed previously (Yamane, 2018; Ghosn et al., 2019). Finally, the existence of EMP-derived NK cells, possessing a potent degranulation response, has been reported recently (Dege et al., 2020). This is particularly striking as NK cells are considered to be of lymphoid origin. However, it is unclear whether these EMP-derived NK cells are part of the myeloid lineage or if these findings have revealed lymphoid potential in EMP. Similarly, it is not clear to what extent EMP-derived NK cells persist into adulthood (Sun et al., 2011; Wu et al., 2014; Corat et al., 2017; Schlums et al., 2017).

## HSC-Independent Macrophages Participate in Embryonic Organogenesis

The role of macrophages in tissue remodeling is an exciting field of ongoing research (Hoeffel and Ginhoux, 2018; Wittamer and Bertrand, 2020). The discovery of adult YS-derived TRM populations with an M2-like non-inflammatory phenotype, associated with wound healing and tissue repair, hints at potential roles in embryonic organogenesis (Takahashi et al., 1998; Rae et al., 2007; Pucci et al., 2009; Fantin et al., 2010; DeFalco et al., 2014; Italiani and Boraschi, 2014; Munro et al., 2019; Shigeta et al., 2019). However, identifying unique and specific roles of HSC-independent cells is complicated by the fact that embryonic organ development spans across all hematopoietic waves. Below we highlight the instances where specific roles for HSC-independent macrophages have been identified (Figure 1B).

### HSC-Independent Embryonic Macrophages Guide Vascular Network Organization in Developing Organs

Vascular networks are established by tip- and stalk- endothelial cells. Endothelial tip-cells, guided by vascular endothelial growth factor (VEGF) gradients, drive the direction of the vessel while the endothelial stalk-cells follow and establish the vessel lumen (Herbert and Stainier, 2011). During embryonic organogenesis, macrophages have been shown to play an essential role in organizing endothelial networks. A role for HSC-independent macrophages in blood vessel anastomosis was first described in detail in the mouse embryonic hindbrain where it is entirely dependent on pMP/early EMP-derived macrophages (Fantin et al., 2010; Rymo et al., 2011). These macrophages invade the brain in a CSF1-dependent manner. Subsequently, upon brain vascularization, the macrophages closely associate with tip-endothelial cells. Macrophage depletion in the brain [*Pu.1* (Scott et al., 1994; McKercher et al., 1996) and *Csf1* KO (Wiktor-Jedrzejczak et al., 1990; Cecchini et al., 1994)], but not specific depletion of FL-derived macrophages (*Lysm*-Cre-mediated diphtheria toxin) (Clausen et al., 1999), significantly reduces the number of vessel intersections and thereby limits the overall complexity of the brain vascular network (Fantin et al., 2010; Rymo et al., 2011).

Hematopoietic stem cells-independent macrophages also play a role in kidney and testis vascular network formation. In mouse embryos, the mesonephros (a temporary kidney structure) and the gonads (which will give rise to the testis in males) are established near the extending nephric ducts around E9. Further extension of the nephric duct results in the generation of uretic buds and the metanephros (precursor to the adult kidneys) around E10-E11.5 (Takasato and Little, 2015). Proliferating primitive pMP/early EMP-derived macrophages (E7.5 pulsed *Csf1r-*Mer-iCre-Mer) are first detected in the gonadal region around E10.5 (DeFalco et al., 2014). By E11.5-E13.5, these macrophages closely associate with and engulf EC of the mesonephros vascular plexus and testis vasculature. Depletion of fetal macrophages (*Cx3cr1*-Cre-mediated diphtheria toxin) results in an enlarged mesonephros vascular plexus, reduced migration of EC into the testis, and impaired development of the coelomic artery. Blood vessels start entering the metanephros between E11.5-E12, and YS-derived macrophages are consistently found perivascular at developing vascular fronts (Rae et al., 2007; Hoeffel et al., 2015; Munro et al., 2019). Analysis of macrophage depleted E12.5 kidney explants (anti-CSF1R depletion) showed increased numbers of unconnected endothelial structures and a reduction in vascular network size, consistent with a role for macrophages in vessel anastomosis.

The developing heart harbors macrophages derived from both the HSC-independent and HSC-dependent hematopoietic waves (*Ccr2*-GFP, *Cx3cr1*-GFP) (Leid et al., 2016). HSC-independent macrophages (E7.5 pulsed *Rosa26-tdCsf1r*-MerCre) appear in the heart around E12.5 and predominantly populate the myocardium where they accumulate near perfused coronary vessels. Genetic depletion of macrophages (*Csf1*^*op/op*^) results in retarded primitive coronary plexus development. However, specific depletion of HSC-derived macrophages (*Ccr2* KO) does not affect primitive coronary plexus development, indicating that HSC-independent macrophages are responsible for the modulation of the myocardial vascular network.

Finally, recent RNA-seq of a human Hofbauer cells (Zulu et al., 2019), a fetus-derived macrophage population found in the placenta, suggests that they may play a role in angiogenesis and remodeling (Thomas et al., 2021). Although Hofbauer cells have also been identified in mice (Takahashi et al., 1991), their role has not yet been investigated *in vivo*.

It is tempting to postulate a generalized role for HSC-independent macrophages in the establishment of vascular networks during embryonic organogenesis. In support of this, HSC-independent macrophages have a similar role in organizing vascular networks in zebrafish, independently of specific organs (Fantin et al., 2010).

### HSC-Independent Macrophages Directly Support Organogenesis

Hematopoietic stem cells-independent macrophages have also been directly implicated in embryonic organ development. The central nervous system is arguably one of the best-studied systems in this context, with YS-derived microglia playing a role in multiple perinatal brain developmental events (Li and Barres, 2018; Low and Ginhoux, 2018). Around E14.5, microglia accumulate near developing axonal tracts and their genetic (*Pu.1* KO)/antibody-based depletion (anti-CSF1R) or their inappropriate activation (E13.5 lipopolysaccharide maternal immune activation), affects the development of interneuronal connections and dopaminergic axon outgrowth (Squarzoni et al., 2014). Similarly, in the peripheral nervous system, macrophages/microglia are found in close contact with developing sensory neurons in dorsal root ganglia from E11. Genetic (*Pu.1* KO) or antibody-based depletion (anti-CSF1R) of these macrophages alters the developmental trajectory of the sensory neurons (Angelim et al., 2018).

During gonad development, macrophages associate with and engulf mislocated germ (E10.5–E11.5) and Sertoli (E12.5) cells, and their absence (*Cx3cr1*-Cre/diphtheria toxin) results in irregularly branched and shortened testis cords (DeFalco et al., 2014). Furthermore, depletion of macrophages during embryogenesis (*Csf1*^*op/op*^, anti-CSF1R depletion) but not postnatally (*Ccr2* KO, anti-CSF1 depletion at birth) results in impaired spermatogenesis after birth (Lokka et al., 2020). Similar observations have been made in kidney development where the clearance of rostral nephrogenic cells and uretic bud formation are delayed in the absence of YS-macrophages (*Cx3cr1*-Cre/diphtheria toxin) (Munro et al., 2019). These results suggest that embryonic macrophages are participating in gonad/testis and kidney development.

Yolk sac-derived osteoclasts are essential for normal skeletal development in the embryo and their absence (*Csf1r* KO model and *Csf1*-Cre-mediated *Tnfrsf11a* KO) disrupts tooth eruption, skull formation, long bone formation, and their timely hematopoietic colonization (Yoshida et al., 1990; Dougall et al., 1999; Dai et al., 2002; Jacome-Galarza et al., 2019). This phenotype is not observed when HSC-derived macrophages are deleted (*Flt3*-Cre-mediated and *Csf1r* KO) (Jacome-Galarza et al., 2019).

Finally, heart development also depends on HSC-independent macrophages which, interestingly, originate locally from HE cells populating the endocardium (Nakano et al., 2013; Yzaguirre and Speck, 2016; Shigeta et al., 2019). An important phase in heart development is the establishment and remodeling of the heart valves which starts around E9.5 and concludes after birth. Specific depletion of endocardial macrophages (*Nfatc*-Cre-mediated *Csf1r* KO) demonstrated that they are essential for heart valve development and that macrophages of other sources cannot compensate for their loss (Shigeta et al., 2019).

### HSC-Independent Macrophages Support HSC Formation

Arguably the most striking function of HSC-independent macrophages is that they can affect HSC ontogeny. This has been studied in detail in zebrafish. HSCs generated in the dorsal aorta of zebrafish enter the circulation via the postcardinal vein (PCV) (Bertrand et al., 2010; Kissa and Herbomel, 2010; Lam et al., 2010). This requires newly formed HSC to traverse the mesenchyme separating the two vessels. Primitive macrophages accumulate in this subaortic mesenchyme and, via metalloproteinases mediated extracellular matrix degradation, create tracks for the HSC to enter the subaortic mesenchyme. These primitive macrophages then join the PCV from where they migrate to the zebrafish FL equivalent, known as the caudal hematopoietic tissue (CHT; Travnickova et al., 2015). Once the HSC reaches the CHT, a specific set of primitive VCAM+ macrophages (usher macrophages) interact with and “capture” passing HSPC and guide them into the CHT (Li D. et al., 2018).

In mice, HSC-independent macrophages also play an important role in HSC ontogeny. At E10.5, HSC-independent macrophages are found in close association with EC and IAHC in the AGM, where they possibly participate in moving KIT+ IAHC cells towards the aortic lumen (Mariani et al., 2019). The CXCL3 chemokine (expressed amongst others by HE and IAHC) is important for the macrophage accumulation in the AGM (Mariani et al., 2019). The deletion of its receptor, *Cxcr3*, results in increased numbers of macrophages in the YS and reduced numbers in the AGM. Reduction of the number of macrophages in the AGM, either by genetic (*Cxcr3* KO) or chemical (clodronate and CSF1R inhibitor BLZ945) depletion, negatively affects HSC generation in the AGM (Mariani et al., 2019). Furthermore, both direct and indirect (transwell) co-culture experiments of AGM-derived aortic endothelial cells (including HE) with aortic macrophages result in an increase of the hematopoietic colony-forming capacity of the endothelial cells (Mariani et al., 2019). These results indicate an essential role for macrophage secreted factors in AGM EHT. RNA-seq of the aortic macrophages revealed that despite having an immune phenotype associated with anti-inflammatory or M2 type phenotype, they have a distinct pro-inflammatory transcriptome (Mariani et al., 2019). Currently, it is unclear if these macrophages promote emergence of all, or only subsets, of HSC.

## Concluding Remarks

In the last decade, it has become clear that HSC-independent hematopoietic cells have previously unanticipated roles in both embryos and adults. They have been found to participate in organogenesis and persist in adults as distinct hematopoietic populations. There are however still many open questions about their exact role, origin, and contributions. In this context, the development of more precise and efficient genetic tracing models would be beneficial. Specifically, models that can efficiently differentiate wave 2 (late-EMP and LMP) from wave 3 (HSC) are needed.

It is also essential to acquire more detailed knowledge of the different hematopoietic waves, both mechanistically and in terms of their exact sites of origin. Indeed, the observation that heart-specific HE can give rise to a specialized population of macrophages, raises the question whether other specialized hematopoietic cells are produced in a site or organ-specific way. Both in the AGM and the YS, multiple sites of hematopoietic emergence have been described (Muller et al., 1994; Medvinsky and Dzierzak, 1996; de Bruijn et al., 2000; Li et al., 2012; Frame et al., 2016; Kasaai et al., 2017). Closer investigation of these known sites as well as the identification of new sites could reveal the existence of new, functionally unique, hematopoietic populations. Furthermore, understanding if and how the distinct hematopoietic cells generated by the different waves interact to optimize blood production is equally fascinating. Altogether such knowledge could provide cues to develop better strategies for *in vitro* generation of HSCs and/or specific blood lineages from embryonic- and induced pluripotent stem cells (ES and IPSC). IPSC generated from different cellular sources may be inherently primed towards specific hematopoietic lineages. Additionally, it may be beneficial to incorporate mature hematopoietic cells into *in vitro* blood production protocols. Along these lines, it has been recently reported that macrophages can support the *in vitro* production of mature enucleated erythroid cells (Lopez-Yrigoyen et al., 2019).

Finally, findings in animal model systems are starting to be confirmed in humans. Macrophages have been found to accumulate in the human AGM at the time of HSC formation (Travnickova et al., 2015), and single-cell sequencing indicates that human microglia are also derived from HSC-independent hematopoietic waves (Bian et al., 2020). In conclusion, it is now well established that HSC-independent hematopoiesis is essential for embryonic organogenesis and its progeny can, and does, persist after birth. This has opened up a new and fascinating field of hematopoietic research.

## Author Contributions

WHN and ML wrote the manuscript. MZHF produced the figure. GL revised and edited the manuscript. All authors approved the final manuscript.

## Conflict of Interest

The authors declare that the research was conducted in the absence of any commercial or financial relationships that could be construed as a potential conflict of interest.

## References

[B1] Adolfsson J. Mansson R. Buza-Vidas N. Hultquist A. Liuba K. Jensen C. T. (2005). Identification of Flt3+ lympho-myeloid stem cells lacking erythro-megakaryocytic potential a revised road map for adult blood lineage commitment. *Cell* 121 295–306. 10.1016/j.cell.2005.02.013 15851035

[B2] Ajami B. Bennett J. L. Krieger C. Tetzlaff W. Rossi F. M. (2007). Local self-renewal can sustain CNS microglia maintenance and function throughout adult life. *Nat. Neurosci.* 10 1538–1543. 10.1038/nn2014 18026097

[B3] Amaya E. (2013). The hemangioblast: a state of competence. *Blood* 122 3853–3854. 10.1182/blood-2013-10-533075 24311714

[B4] Angelim M. Maia L. Mouffle C. Ginhoux F. Low D. Amancio-Dos-Santos A. (2018). Embryonic macrophages and microglia ablation alter the development of dorsal root ganglion sensory neurons in mouse embryos. *Glia* 66 2470–2486. 10.1002/glia.23499 30252950

[B5] Azzoni E. Frontera V. McGrath K. E. Harman J. Carrelha J. Nerlov C. (2018). Kit ligand has a critical role in mouse yolk sac and aorta-gonad-mesonephros hematopoiesis. *EMBO Rep.* 19:, e45477. 10.15252/embr.201745477 PMC6172468 30166337

[B6] Benz C. Martins V. C. Radtke F. Bleul C. C. (2008). The stream of precursors that colonizes the thymus proceeds selectively through the early T lineage precursor stage of T cell development. *J. Exp. Med.* 205 1187–1199. 10.1084/jem.20072168 18458114 PMC2373849

[B7] Bertrand J. Y. Chi N. C. Santoso B. Teng S. Stainier D. Y. Traver D. (2010). Haematopoietic stem cells derive directly from aortic endothelium during development. *Nature* 464 108–111. 10.1038/nature08738 20154733 PMC2858358

[B8] Bhowmick N. A. Chytil A. Plieth D. Gorska A. E. Dumont N. Shappell S. (2004). TGF-beta signaling in fibroblasts modulates the oncogenic potential of adjacent epithelia. *Science* 303 848–851. 10.1126/science.1090922 14764882

[B9] Bian Z. Gong Y. Huang T. Lee C. Z. W. Bian L. Bai Z. (2020). Deciphering human macrophage development at single-cell resolution. *Nature* 582 571–576. 10.1038/s41586-020-2316-7 32499656

[B10] Boiers C. Carrelha J. Lutteropp M. Luc S. Green J. C. Azzoni E. (2013). Lymphomyeloid contribution of an immune-restricted progenitor emerging prior to definitive hematopoietic stem cells. *Cell Stem Cell* 13 535–548. 10.1016/j.stem.2013.08.012 24054998

[B11] Boisset J. C. van Cappellen W. Andrieu-Soler C. Galjart N. Dzierzak E. Robin C. (2010). In vivo imaging of haematopoietic cells emerging from the mouse aortic endothelium. *Nature* 464 116–120. 10.1038/nature08764 20154729

[B12] Boring L. Gosling J. Chensue S. W. Kunkel S. L. Farese R. V. Jr. Broxmeyer H. E. (1997). Impaired monocyte migration and reduced type 1 (Th1) cytokine responses in C-C chemokine receptor 2 knockout mice. *J. Clin. Invest.* 100 2552–2561. 10.1172/jci119798 9366570 PMC508456

[B13] Cecchini M. G. Dominguez M. G. Mocci S. Wetterwald A. Felix R. Fleisch H. (1994). Role of colony stimulating factor-1 in the establishment and regulation of tissue macrophages during postnatal development of the mouse. *Development* 120 1357–1372. 8050349 10.1242/dev.120.6.1357

[B14] Chen M. J. Li Y. De Obaldia M. E. Yang Q. Yzaguirre A. D. Yamada-Inagawa T. (2011). Erythroid/myeloid progenitors and hematopoietic stem cells originate from distinct populations of endothelial cells. *Cell Stem Cell* 9 541–552. 10.1016/j.stem.2011.10.003 22136929 PMC3576591

[B15] Chen M. J. Yokomizo T. Zeigler B. M. Dzierzak E. Speck N. A. (2009). Runx1 is required for the endothelial to haematopoietic cell transition but not thereafter. *Nature* 457 887–891. 10.1038/nature07619 19129762 PMC2744041

[B16] Clausen B. E. Burkhardt C. Reith W. Renkawitz R. Forster I. (1999). Conditional gene targeting in macrophages and granulocytes using LysMcre mice. *Transgenic. Res.* 8 265–277. 10621974 10.1023/a:1008942828960

[B17] Corat M. A. Schlums H. Wu C. Theorell J. Espinoza D. A. Sellers S. E. (2017). Acquired somatic mutations in PNH reveal long-term maintenance of adaptive NK cells independent of HSPCs. *Blood* 129 1940–1946. 10.1182/blood-2016-08-734285 27903532 PMC5383870

[B18] Dai X. M. Ryan G. R. Hapel A. J. Dominguez M. G. Russell R. G. Kapp S. (2002). Targeted disruption of the mouse colony-stimulating factor 1 receptor gene results in osteopetrosis, mononuclear phagocyte deficiency, increased primitive progenitor cell frequencies, and reproductive defects. *Blood* 99 111–120. 10.1182/blood.v99.1.111 11756160

[B19] Davidson A. J. Zon L. I. (2000). Turning mesoderm into blood: the formation of hematopoietic stem cells during embryogenesis. *Curr. Top Dev. Biol.* 50 45–60. 10.1016/s0070-2153(00)50003-9 10948449

[B20] de Bruijn M. F. Speck N. A. Peeters M. C. Dzierzak E. (2000). Definitive hematopoietic stem cells first develop within the major arterial regions of the mouse embryo. *EMBO J.* 19 2465–2474. 10.1093/emboj/19.11.2465 10835345 PMC212758

[B21] DeFalco T. Bhattacharya I. Williams A. V. Sams D. M. Capel B. (2014). Yolk-sac-derived macrophages regulate fetal testis vascularization and morphogenesis. *Proc. Natl. Acad. Sci. U. S. A.* 111 E2384–E2393. 24912173 10.1073/pnas.1400057111 PMC4060703

[B22] Dege C. Fegan K. H. Creamer J. P. Berrien-Elliott M. M. Luff S. A. Kim D. (2020). Potently cytotoxic natural killer cells initially emerge from Erythro-myeloid progenitors during mammalian development. *Dev. Cell* 53 229–239e7. 32197069 10.1016/j.devcel.2020.02.016 PMC7185477

[B23] Ding L. Saunders T. L. Enikolopov G. Morrison S. J. (2012). Endothelial and perivascular cells maintain haematopoietic stem cells. *Nature* 481 457–462. 10.1038/nature10783 22281595 PMC3270376

[B24] Dougall W. C. Glaccum M. Charrier K. Rohrbach K. Brasel K. De Smedt T. (1999). RANK is essential for osteoclast and lymph node development. *Genes Dev.* 13 2412–2424. 10.1101/gad.13.18.2412 10500098 PMC317030

[B25] Dzierzak E. Bigas A. (2018). Blood development: hematopoietic stem cell dependence and independence. *Cell Stem Cell* 22 639–651. 10.1016/j.stem.2018.04.015 29727679

[B26] Easley-Neal C. Foreman O. Sharma N. Zarrin A. A. Weimer R. M. (2019). CSF1R Ligands IL-34 and CSF1 are differentially required for microglia development and maintenance in white and Gray matter brain regions. *Front. Immunol.* 10:2199. 10.3389/fimmu.2019.02199 31616414 PMC6764286

[B27] Eilken H. M. Nishikawa S. Schroeder T. (2009). Continuous single-cell imaging of blood generation from haemogenic endothelium. *Nature* 457 896–900. 10.1038/nature07760 19212410

[B28] Epelman S. Lavine K. J. Beaudin A. E. Sojka D. K. Carrero J. A. Calderon B. (2014). Embryonic and adult-derived resident cardiac macrophages are maintained through distinct mechanisms at steady state and during inflammation. *Immunity* 40 91–104. 10.1016/j.immuni.2013.11.019 24439267 PMC3923301

[B29] Fantin A. Vieira J. M. Gestri G. Denti L. Schwarz Q. Prykhozhij S. (2010). Tissue macrophages act as cellular chaperones for vascular anastomosis downstream of VEGF-mediated endothelial tip cell induction. *Blood* 116 829–840. 10.1182/blood-2009-12-257832 20404134 PMC2938310

[B30] Feil R. Wagner J. Metzger D. Chambon P. (1997). Regulation of Cre recombinase activity by mutated estrogen receptor ligand-binding domains. *Biochem. Biophys. Res. Commun.* 237 752–757. 10.1006/bbrc.1997.7124 9299439

[B31] Frame J. M. Fegan K. H. Conway S. J. McGrath K. E. Palis J. (2016). Definitive hematopoiesis in the yolk sac emerges from Wnt-responsive hemogenic endothelium independently of circulation and arterial identity. *Stem Cells* 34 431–444. 10.1002/stem.2213 26418893 PMC4755868

[B32] Garcia-Alegria E. Menegatti S. Fadlullah M. Z. H. Menendez P. Lacaud G. Kouskoff V. (2018). Early human hemogenic endothelium generates primitive and definitive hematopoiesis *In Vitro*. *Stem Cell Rep.orts* 11 1061–1074. 10.1016/j.stemcr.2018.09.013 30449319 PMC6234921

[B33] Gentek R. Ghigo C. Hoeffel G. Bulle M. J. Msallam R. Gautier G. (2018a). Hemogenic endothelial fate mapping reveals dual developmental origin of mast cells. *Immunity* 48 1160–1171e5. 29858009 10.1016/j.immuni.2018.04.025

[B34] Gentek R. Ghigo C. Hoeffel G. Jorquera A. Msallam R. Wienert S. (2018b). Epidermal gammadelta T cells originate from yolk sac hematopoiesis and clonally self-renew in the adult. *J. Exp. Med.* 215 2994–3005. 10.1084/jem.20181206 30409784 PMC6279412

[B35] Ghosn E. Yoshimoto M. Nakauchi H. Weissman I. L. Herzenberg L. A. (2019). Hematopoietic stem cell-independent hematopoiesis and the origins of innate-like B lymphocytes. *Development* 146 dev170571. 10.1242/dev.170571 31371526 PMC6703711

[B36] Ginhoux F. Greter M. Leboeuf M. Nandi S. See P. Gokhan S. (2010). Fate mapping analysis reveals that adult microglia derive from primitive macrophages. *Science* 330 841–845. 10.1126/science.1194637 20966214 PMC3719181

[B37] Ginhoux F. Guilliams M. (2016). Tissue-resident macrophage ontogeny and homeostasis. *Immunity* 44 439–449. 10.1016/j.immuni.2016.02.024 26982352

[B38] Gomez Perdiguero E. Klapproth K. Schulz C. Busch K. Azzoni E. Crozet L. (2015). Tissue-resident macrophages originate from yolk-sac-derived erythro-myeloid progenitors. *Nature* 518 547–551. 10.1038/nature13989 25470051 PMC5997177

[B39] Gouon-Evans V. Rothenberg M. E. Pollard J. W. (2000). Postnatal mammary gland development requires macrophages and eosinophils. *Development* 127 2269–2282. 10804170 10.1242/dev.127.11.2269

[B40] Greter M. Lelios I. Pelczar P. Hoeffel G. Price J. Leboeuf M. (2012). Stroma-derived interleukin-34 controls the development and maintenance of langerhans cells and the maintenance of microglia. *Immunity* 37 1050–1060. 10.1016/j.immuni.2012.11.001 23177320 PMC4291117

[B41] Guilliams M. De Kleer I. Henri S. Post S. Vanhoutte L. De Prijck S. (2013). Alveolar macrophages develop from fetal monocytes that differentiate into long-lived cells in the first week of life via GM-CSF. *J. Exp. Med.* 210 1977–1992. 10.1084/jem.20131199 24043763 PMC3782041

[B42] Hagemeyer N. Kierdorf K. Frenzel K. Xue J. Ringelhan M. Abdullah Z. (2016). Transcriptome-based profiling of yolk sac-derived macrophages reveals a role for Irf8 in macrophage maturation. *EMBO J.* 35 1730–1744. 10.15252/embj.201693801 27412700 PMC5010043

[B43] Hanna R. N. Carlin L. M. Hubbeling H. G. Nackiewicz D. Green A. M. Punt J. A. (2011). The transcription factor NR4A1 (Nur77) controls bone marrow differentiation and the survival of Ly6C- monocytes. *Nat. Immunol.* 12 778–785. 10.1038/ni.2063 21725321 PMC3324395

[B44] Hashimoto D. Chow A. Noizat C. Teo P. Beasley M. B. Leboeuf M. (2013). Tissue-resident macrophages self-maintain locally throughout adult life with minimal contribution from circulating monocytes. *Immunity* 38 792–804. 10.1016/j.immuni.2013.04.004 23601688 PMC3853406

[B45] Hassnain Waqas S. F. Noble A. Hoang A. C. Ampem G. Popp M. Strauss S. (2017). Adipose tissue macrophages develop from bone marrow-independent progenitors in Xenopus laevis and mouse. *J. Leukoc. Biol.* 102 845–855. 10.1189/jlb.1a0317-082rr 28642277 PMC5574031

[B46] Herbert S. P. Stainier D. Y. (2011). Molecular control of endothelial cell behaviour during blood vessel morphogenesis. *Nat. Rev. Mol. Cell Biol.* 12 551–564. 10.1038/nrm3176 21860391 PMC3319719

[B47] Hoeffel G. Chen J. Lavin Y. Low D. Almeida F. F. See P. (2015). C-Myb(+) erythro-myeloid progenitor-derived fetal monocytes give rise to adult tissue-resident macrophages. *Immunity* 42 665–678. 10.1016/j.immuni.2015.03.011 25902481 PMC4545768

[B48] Hoeffel G. Ginhoux F. (2018). Fetal monocytes and the origins of tissue-resident macrophages. *Cell Immunol.* 330 5–15. 10.1016/j.cellimm.2018.01.001 29475558

[B49] Hoeffel G. Wang Y. Greter M. See P. Teo P. Malleret B. (2012). Adult Langerhans cells derive predominantly from embryonic fetal liver monocytes with a minor contribution of yolk sac-derived macrophages. *J. Exp. Med.* 209 1167–1181. 10.1084/jem.20120340 22565823 PMC3371735

[B50] Hoess R. H. Abremski K. (1984). Interaction of the bacteriophage P1 recombinase Cre with the recombining site loxP. *Proc. Natl. Acad. Sci. U. S. A.* 81 1026–1029. 10.1073/pnas.81.4.1026 6230671 PMC344756

[B51] Ide S. Yahara Y. Kobayashi Y. Strausser S. A. Ide K. Watwe A. (2020). Yolk-sac-derived macrophages progressively expand in the mouse kidney with age. *Elife* 9:, e51756. 10.7554/eLife.51756 PMC7205460 32301704

[B52] Imai T. Hieshima K. Haskell C. Baba M. Nagira M. Nishimura M. (1997). Identification and molecular characterization of fractalkine receptor CX3CR1, which mediates both leukocyte migration and adhesion. *Cell* 91 521–530. 10.1016/s0092-8674(00)80438-9 9390561

[B53] Italiani P. Boraschi D. (2014). From monocytes to M1/M2 macrophages: phenotypical vs. functional differentiation. *Front. Immunol.* 5:514. 10.3389/fimmu.2014.00514 25368618 PMC4201108

[B54] Iwasaki H. Somoza C. Shigematsu H. Duprez E. A. Iwasaki-Arai J. Mizuno S. (2005). Distinctive and indispensable roles of PU.1 in maintenance of hematopoietic stem cells and their differentiation. *Blood* 106 1590–1600. 10.1182/blood-2005-03-0860 15914556 PMC1895212

[B55] Jacome-Galarza C. E. Percin G. I. Muller J. T. Mass E. Lazarov T. Eitler J. (2019). Developmental origin, functional maintenance and genetic rescue of osteoclasts. *Nature* 568 541–545. 10.1038/s41586-019-1105-7 30971820 PMC6910203

[B56] Jacquelin S. Licata F. Dorgham K. Hermand P. Poupel L. Guyon E. (2013). CX3CR1 reduces Ly6Chigh-monocyte motility within and release from the bone marrow after chemotherapy in mice. *Blood* 122 674–683. 10.1182/blood-2013-01-480749 23775714

[B57] Jaffredo T. Gautier R. Eichmann A. Dieterlen-Lièvre F. (1998). Intraaortic hemopoietic cells are derived from endothelial cells during ontogeny. *Development* 22, 4574–4583. 10.1242/dev.125.22.4575 9778515

[B58] Jaitin D. A. Adlung L. Thaiss C. A. Weiner A. Li B. Descamps H. (2019). Lipid-associated macrophages control metabolic homeostasis in a Trem2-dependent manner. *Cell* 178 686–698e14. 31257031 10.1016/j.cell.2019.05.054 PMC7068689

[B59] Jakubzick C. Gautier E. L. Gibbings S. L. Sojka D. K. Schlitzer A. Johnson T. E. (2013). Minimal differentiation of classical monocytes as they survey steady-state tissues and transport antigen to lymph nodes. *Immunity* 39 599–610. 10.1016/j.immuni.2013.08.007 24012416 PMC3820017

[B60] Jappinen N. Felix I. Lokka E. Tyystjarvi S. Pynttari A. Lahtela T. (2019). Fetal-derived macrophages dominate in adult mammary glands. *Nat. Commun.* 10:, 281. 10.1038/s41467-018-08065-1 PMC6336770 30655530

[B61] Jokela H. Lokka E. Kiviranta M. Tyystjarvi S. Gerke H. Elima K. (2020). Fetal-derived macrophages persist and sequentially maturate in ovaries after birth in mice. *Eur. J. Immunol.* 50 1500–1514. 10.1002/eji.202048531 32459864

[B62] Jung S. Aliberti J. Graemmel P. Sunshine M. J. Kreutzberg G. W. Sher A. (2000). Analysis of fractalkine receptor CX(3)CR1 function by targeted deletion and green fluorescent protein reporter gene insertion. *Mol. Cell Biol.* 20 4106–4114. 10.1128/mcb.20.11.4106-4114.2000 10805752 PMC85780

[B63] Kasaai B. Caolo V. Peacock H. M. Lehoux S. Gomez-Perdiguero E. Luttun A. (2017). Erythro-myeloid progenitors can differentiate from endothelial cells and modulate embryonic vascular remodeling. *Sci. Rep.* 7:, 43817. 10.1038/srep43817 PMC5341067 28272478

[B64] Kierdorf K. Erny D. Goldmann T. Sander V. Schulz C. Perdiguero E. G. (2013). Microglia emerge from erythromyeloid precursors via Pu.1- and Irf8-dependent pathways. *Nat. Neurosci.* 16 273–280. 10.1038/nn.3318 23334579

[B65] Kim H. G. de Guzman C. G. Swindle C. S. Cotta C. V. Gartland L. Scott E. W. (2004). The ETS family transcription factor PU.1 is necessary for the maintenance of fetal liver hematopoietic stem cells. *Blood* 104 3894–3900. 10.1182/blood-2002-08-2425 15328162

[B66] Kishimoto I. Okano T. Nishimura K. Motohashi T. Omori K. (2019). Early development of resident macrophages in the mouse cochlea depends on yolk sac hematopoiesis. *Front. Neurol.* 10:1115. 10.3389/fneur.2019.01115 31695671 PMC6817595

[B67] Kissa K. Herbomel P. (2010). Blood stem cells emerge from aortic endothelium by a novel type of cell transition. *Nature* 464 112–115. 10.1038/nature08761 20154732

[B68] Kitamura Y. Go S. Hatanaka K. (1978). Decrease of mast cells in W/Wv mice and their increase by bone marrow transplantation. *Blood* 52 447–452. 10.1182/blood.v52.2.447.bloodjournal522447 352443

[B69] Kitamura Y. Shimada M. Hatanaka K. Miyano Y. (1977). Development of mast cells from grafted bone marrow cells in irradiated mice. *Nature* 268 442–443. 10.1038/268442a0 331117

[B70] Kobayashi M. Shelley W. C. Seo W. Vemula S. Lin Y. Liu Y. (2014). Functional B-1 progenitor cells are present in the hematopoietic stem cell-deficient embryo and depend on Cbfbeta for their development. *Proc. Natl. Acad. Sci. U. S. A.* 111 12151–12156. 10.1073/pnas.1407370111 25092306 PMC4143017

[B71] Kondo Y. Duncan I. D. (2009). Selective reduction in microglia density and function in the white matter of colony-stimulating factor-1-deficient mice. *J. Neurosci. Res.* 87 2686–2695. 10.1002/jnr.22096 19396881 PMC4843845

[B72] Lacaud G. Kouskoff V. (2017). Hemangioblast, hemogenic endothelium, and primitive versus definitive hematopoiesis. *Exp. Hematol.* 49 19–24. 10.1016/j.exphem.2016.12.009 28043822

[B73] Lam E. Y. Hall C. J. Crosier P. S. Crosier K. E. Flores M. V. (2010). Live imaging of Runx1 expression in the dorsal aorta tracks the emergence of blood progenitors from endothelial cells. *Blood* 116 909–914. 10.1182/blood-2010-01-264382 20453160

[B74] Lancrin C. Sroczynska P. Stephenson C. Allen T. Kouskoff V. Lacaud G. (2009). The haemangioblast generates haematopoietic cells through a haemogenic endothelium stage. *Nature* 457 892–895. 10.1038/nature07679 19182774 PMC2661201

[B75] Lee S. L. Wesselschmidt R. L. Linette G. P. Kanagawa O. Russell J. H. Milbrandt J. (1995). Unimpaired thymic and peripheral T cell death in mice lacking the nuclear receptor NGFI-B (Nur77). *Science* 269 532–535. 10.1126/science.7624775 7624775

[B76] Lehnert B. E. Valdez Y. E. Holland L. M. (1985). Pulmonary macrophages: alveolar and interstitial populations. *Exp. Lung. Res.* 9 177–190. 10.3109/01902148509057522 3000757

[B77] Leid J. Carrelha J. Boukarabila H. Epelman S. Jacobsen S. E. Lavine K. J. (2016). Primitive embryonic macrophages are required for coronary development and maturation. *Circ. Res.* 118 1498–1511. 10.1161/circresaha.115.308270 27009605 PMC5567774

[B78] Li D. Xue W. Li M. Dong M. Wang J. Wang X. (2018). VCAM-1(+) macrophages guide the homing of HSPCs to a vascular niche. *Nature* 564 119–124. 10.1038/s41586-018-0709-7 30455424 PMC6492262

[B79] Li F. Okreglicka K. M. Pohlmeier L. M. Schneider C. Kopf M. (2020). Fetal monocytes possess increased metabolic capacity and replace primitive macrophages in tissue macrophage development. *EMBO J.* 39:, e103205. 10.15252/embj.2019103205 PMC6996567 31894879

[B80] Li Q. Barres B. A. (2018). Microglia and macrophages in brain homeostasis and disease. *Nat. Rev. Immunol.* 18 225–242. 10.1038/nri.2017.125 29151590

[B81] Li Z. Lan Y. He W. Chen D. Wang J. Zhou F. (2012). Mouse embryonic head as a site for hematopoietic stem cell development. *Cell Stem Cell* 11 663–675. 10.1016/j.stem.2012.07.004 23122290

[B82] Li Z. Liu S. Xu J. Zhang X. Han D. Liu J. (2018). Adult connective tissue-resident mast cells originate from late Erythro-myeloid progenitors. *Immunity* 49 640–653e5. 30332630 10.1016/j.immuni.2018.09.023

[B83] Liegeois M. Legrand C. Desmet C. J. Marichal T. Bureau F. (2018). The interstitial macrophage: a long-neglected piece in the puzzle of lung immunity. *Cell Immunol.* 330 91–96. 10.1016/j.cellimm.2018.02.001 29458975

[B84] Liu Z. Gu Y. Chakarov S. Bleriot C. Kwok I. Chen X. (2019). Fate mapping via Ms4a3-expression history traces monocyte-derived cells. *Cell* 178 1509–1525e19. 31491389 10.1016/j.cell.2019.08.009

[B85] Lokka E. Lintukorpi L. Cisneros-Montalvo S. Makela J. A. Tyystjarvi S. Ojasalo V. (2020). Generation, localization and functions of macrophages during the development of testis. *Nat. Commun.* 11:, 4375. 10.1038/s41467-020-18206-0 PMC7463013 32873797

[B86] Lopez-Yrigoyen M. Yang C. T. Fidanza A. Cassetta L. Taylor A. H. McCahill A. (2019). Genetic programming of macrophages generates an *in vitro* model for the human erythroid island niche. *Nat. Commun.* 10:, 881. 10.1038/s41467-019-08705-0 PMC6382809 30787325

[B87] Low D. Ginhoux F. (2018). Recent advances in the understanding of microglial development and homeostasis. *Cell Immunol.* 330 68–78. 10.1016/j.cellimm.2018.01.004 29366562

[B88] Maeda K. Kobayashi Y. Udagawa N. Uehara S. Ishihara A. Mizoguchi T. (2012). Wnt5a-Ror2 signaling between osteoblast-lineage cells and osteoclast precursors enhances osteoclastogenesis. *Nat. Med.* 18 405–412. 10.1038/nm.2653 22344299

[B89] Mariani S. A. Li Z. Rice S. Krieg C. Fragkogianni S. Robinson M. (2019). Pro-inflammatory aorta-associated macrophages are involved in embryonic development of hematopoietic stem cells. *Immunity* 50 1439–1452e5. 10.1016/j.immuni.2019.05.003 31178352 PMC6591003

[B90] Marks S. C. Jr. Lane P. W. (1976). Osteopetrosis, a new recessive skeletal mutation on chromosome 12 of the mouse. *J. Hered* 67 11–18. 10.1093/oxfordjournals.jhered.a108657 1262696

[B91] Mass E. (2018). Delineating the origins, developmental programs and homeostatic functions of tissue-resident macrophages. *Int. Immunol.* 30 493–501. 10.1093/intimm/dxy044 29986024

[B92] Mass E. Ballesteros I. Farlik M. Halbritter F. Gunther P. Crozet L. (2016). Specification of tissue-resident macrophages during organogenesis. *Science* 353:, aaf4238. 10.1126/science.aaf4238 27492475 PMC5066309

[B93] McCormack M. P. Forster A. Drynan L. Pannell R. Rabbitts T. H. (2003). The LMO2 T-cell oncogene is activated via chromosomal translocations or retroviral insertion during gene therapy but has no mandatory role in normal T-cell development. *Mol. Cell Biol.* 23 9003–9013. 10.1128/MCB.23.24.9003-9013.2003 14645513 PMC309712

[B94] McGrath K. E. Frame J. M. Fegan K. H. Bowen J. R. Conway S. J. Catherman S. C. (2015). Distinct sources of hematopoietic progenitors emerge before HSCs and provide functional blood cells in the mammalian embryo. *Cell Rep.* 11 1892–1904. 10.1016/j.celrep.2015.05.036 26095363 PMC4490098

[B95] McKercher S. R. Torbett B. E. Anderson K. L. Henkel G. W. Vestal D. J. Baribault H. (1996). Targeted disruption of the PU.1 gene results in multiple hematopoietic abnormalities. *EMBO J.* 15 5647–5658. 10.1002/j.1460-2075.1996.tb00949.x 8896458 PMC452309

[B96] Medvinsky A. Dzierzak E. (1996). Definitive hematopoiesis is autonomously initiated by the AGM region. *Cell* 86 897–906. 10.1016/S0092-8674(00)80165-8 8808625

[B97] Medvinsky A. L. Samoylina N. L. Muller A. M. Dzierzak E. A. (1993). An early pre-liver intraembryonic source of CFU-S in the developing mouse. *Nature* 364 64–67. 10.1038/364064a0 8316298

[B98] Merad M. Manz M. G. Karsunky H. Wagers A. Peters W. Charo I. (2002). Langerhans cells renew in the skin throughout life under steady-state conditions. *Nat. Immunol.* 3 1135–1141. 10.1038/ni852 12415265 PMC4727838

[B99] Metzger D. Clifford J. Chiba H. Chambon P. (1995). Conditional site-specific recombination in mammalian cells using a ligand-dependent chimeric Cre recombinase. *Proc. Natl. Acad. Sci. U. S. A.* 92 6991–6995. 10.1073/pnas.92.15.6991 7624356 PMC41457

[B100] Miller J. Horner A. Stacy T. Lowrey C. Lian J. B. Stein G. (2002). The core-binding factor beta subunit is required for bone formation and hematopoietic maturation. *Nat. Genet.* 32 645–649. 10.1038/ng1049 12434155

[B101] Mucenski M. L. McLain K. Kier A. B. Swerdlow S. H. Schreiner C. M. Miller T. A. (1991). A functional c-myb gene is required for normal murine fetal hepatic hematopoiesis. *Cell* 65 677–689. 10.1016/0092-8674(91)90099-K 1709592

[B102] Muller A. M. Medvinsky A. Strouboulis J. Grosveld F. Dzierzak E. (1994). Development of hematopoietic stem cell activity in the mouse embryo. *Immunity* 1 291–301. 10.1016/1074-7613(94)90081-7 7889417

[B103] Munro D. A. D. Wineberg Y. Tarnick J. Vink C. S. Li Z. Pridans C. (2019). Macrophages restrict the nephrogenic field and promote endothelial connections during kidney development. *Elife* 8:, e43271. 10.7554/eLife.43271.042 PMC6374076 30758286

[B104] Myers C. T. Krieg P. A. (2013). BMP-mediated specification of the erythroid lineage suppresses endothelial development in blood island precursors. *Blood* 122 3929–3939. 10.1182/blood-2013-03-490045 24100450 PMC3854112

[B105] Nakano H. Liu X. Arshi A. Nakashima Y. van Handel B. Sasidharan R. (2013). Haemogenic endocardium contributes to transient definitive haematopoiesis. *Nat. Commun.* 4:, 1564. 10.1038/ncomms2569 23463007 PMC3612528

[B106] Niki M. Okada H. Takano H. Kuno J. Tani K. Hibino H. (1997). Hematopoiesis in the fetal liver is impaired by targeted mutagenesis of a gene encoding a non-DNA binding subunit of the transcription factor, polyomavirus enhancer binding protein 2/core binding factor. *Proc. Natl. Acad. Sci. U. S. A.* 94 5697–5702. 10.1073/pnas.94.11.5697 9159135 PMC20841

[B107] North T. Gu T. L. Stacy T. Wang Q. Howard L. Binder M. (1999). Cbfa2 is required for the formation of intra-aortic hematopoietic clusters. *Development* 126 2563–2575. 10226014 10.1242/dev.126.11.2563

[B108] Okuda T. van Deursen J. Hiebert S. W. Grosveld G. Downing J. R. (1996). AML1, the target of multiple chromosomal translocations in human leukemia, is essential for normal fetal liver hematopoiesis. *Cell* 84 321–330. 10.1016/S0092-8674(00)80986-1 8565077

[B109] Olson M. C. Scott E. W. Hack A. A. Su G. H. Tenen D. G. Singh H. (1995). PU. 1 is not essential for early myeloid gene expression but is required for terminal myeloid differentiation. *Immunity* 3 703–714. 10.1016/1074-7613(95)90060-8 8777716

[B110] Ottersbach K. (2019). Endothelial-to-haematopoietic transition: an update on the process of making blood. *Biochem. Soc. Trans.* 47 591–601. 10.1042/BST20180320 30902922 PMC6490701

[B111] Palis J. (2016). Hematopoietic stem cell-independent hematopoiesis: emergence of erythroid, megakaryocyte, and myeloid potential in the mammalian embryo. *FEBS Lett.* 590 3965–3974. 10.1002/1873-3468.12459 27790707

[B112] Palis J. Robertson S. Kennedy M. Wall C. Keller G. (1999). Development of erythroid and myeloid progenitors in the yolk sac and embryo proper of the mouse. *Development* 126 5073–5084. 10529424 10.1242/dev.126.22.5073

[B113] Percin G. I. Eitler J. Kranz A. Fu J. Pollard J. W. Naumann R. (2018). CSF1R regulates the dendritic cell pool size in adult mice via embryo-derived tissue-resident macrophages. *Nat. Commun.* 9:, 5279. 10.1038/s41467-018-07685-x 30538245 PMC6290072

[B114] Pucci F. Venneri M. A. Biziato D. Nonis A. Moi D. Sica A. (2009). gene signature shared by tumor-infiltrating Tie2-expressing monocytes, blood “resident” monocytes, and embryonic macrophages suggests common functions and developmental relationships. *Blood* 114 901–914. 10.1182/blood-2009-01-200931 19383967

[B115] Qian B. Z. Li J. Zhang H. Kitamura T. Zhang J. Campion L. R. (2011). CCL2 recruits inflammatory monocytes to facilitate breast-tumour metastasis. *Nature* 475 222–225. 10.1038/nature10138 21654748 PMC3208506

[B116] Rae F. Woods K. Sasmono T. Campanale N. Taylor D. Ovchinnikov D. A. (2007). Characterisation and trophic functions of murine embryonic macrophages based upon the use of a Csf1r-EGFP transgene reporter. *Dev. Biol.* 308 232–246. 10.1016/j.ydbio.2007.05.027 17597598

[B117] Rantakari P. Auvinen K. Jappinen N. Kapraali M. Valtonen J. Karikoski M. (2015). The endothelial protein PLVAP in lymphatics controls the entry of lymphocytes and antigens into lymph nodes. *Nat. Immunol.* 16 386–396. 10.1038/ni.3101 25665101

[B118] Rantakari P. Jappinen N. Lokka E. Mokkala E. Gerke H. Peuhu E. (2016). Fetal liver endothelium regulates the seeding of tissue-resident macrophages. *Nature* 538 392–396. 10.1038/nature19814 27732581

[B119] Russo L. Lumeng C. N. (2018). Properties and functions of adipose tissue macrophages in obesity. *Immunology* 155 407–417. 10.1111/imm.13002 30229891 PMC6230999

[B120] Rymo S. F. Gerhardt H. Wolfhagen Sand F. Lang R. Uv A. Betsholtz C. (2011). A two-way communication between microglial cells and angiogenic sprouts regulates angiogenesis in aortic ring cultures. *PLoS One* 6:e15846. 10.1371/journal.pone.0015846 21264342 PMC3018482

[B121] Samokhvalov I. M. Samokhvalova N. I. Nishikawa S. (2007). Cell tracing shows the contribution of the yolk sac to adult haematopoiesis. *Nature* 446 1056–1061. 10.1038/nature05725 17377529

[B122] Sasaki K. Yagi H. Bronson R. T. Tominaga K. Matsunashi T. Deguchi K. (1996). Absence of fetal liver hematopoiesis in mice deficient in transcriptional coactivator core binding factor beta. *Proc. Natl. Acad. Sci. U. S. A.* 93 12359–12363. 10.1073/pnas.93.22.12359 8901586 PMC37996

[B123] Sauer B. Henderson N. (1988). Site-specific DNA recombination in mammalian cells by the Cre recombinase of bacteriophage P1. *Proc. Natl. Acad. Sci. U. S. A.* 85 5166–5170. 10.1073/pnas.85.14.5166 2839833 PMC281709

[B124] Schlums H. Jung M. Han H. Theorell J. Bigley V. Chiang S. C. (2017). Adaptive NK cells can persist in patients with GATA2 mutation depleted of stem and progenitor cells. *Blood* 129 1927–1939. 10.1182/blood-2016-08-734236 28209719 PMC5383869

[B125] Schulz C. Gomez Perdiguero E. Chorro L. Szabo-Rogers H. Cagnard N. Kierdorf K. (2012). A lineage of myeloid cells independent of Myb and hematopoietic stem cells. *Science* 336 86–90. 10.1126/science.1219179 22442384

[B126] Scott E. W. Simon M. C. Anastasi J. Singh H. (1994). Requirement of transcription factor PU.1 in the development of multiple hematopoietic lineages. *Science* 265 1573–1577. 10.1126/science.8079170 8079170

[B127] Sheng J. Ruedl C. Karjalainen K. (2015). Most tissue-resident macrophages except microglia are derived from fetal hematopoietic stem cells. *Immunity* 43 382–393. 10.1016/j.immuni.2015.07.016 26287683

[B128] Shigeta A. Huang V. Zuo J. Besada R. Nakashima Y. Lu Y. (2019). Endocardially derived macrophages are essential for valvular remodeling. *Dev. Cell* 48 617–630e3. 10.1016/j.devcel.2019.01.021 30799229 PMC6440481

[B129] Sorensen I. Adams R. H. Gossler A. (2009). DLL1-mediated Notch activation regulates endothelial identity in mouse fetal arteries. *Blood* 113 5680–5688. 10.1182/blood-2008-08-174508 19144989

[B130] Squarzoni P. Oller G. Hoeffel G. Pont-Lezica L. Rostaing P. Low D. (2014). Microglia modulate wiring of the embryonic forebrain. *Cell Rep.* 8 1271–1279. 10.1016/j.celrep.2014.07.042 25159150

[B131] Stefanska M. Batta K. Patel R. Florkowska M. Kouskoff V. Lacaud G. (2017). Primitive erythrocytes are generated from hemogenic endothelial cells. *Sci. Rep.* 7:, 6401. 10.1038/s41598-017-06627-9 28743905 PMC5526883

[B132] Stewart T. A. Hughes K. Hume D. A. Davis F. M. (2019). Developmental stage-specific distribution of macrophages in mouse mammary gland. *Front. Cell Dev. Biol.* 7:250. 10.3389/fcell.2019.00250 31709255 PMC6821639

[B133] Sun J. C. Beilke J. N. Bezman N. A. Lanier L. L. (2011). Homeostatic proliferation generates long-lived natural killer cells that respond against viral infection. *J. Exp. Med.* 208 357–368. 10.1084/jem.20100479 21262959 PMC3039854

[B134] Takahashi K. Donovan M. J. Rogers R. A. Ezekowitz R. A. (1998). Distribution of murine mannose receptor expression from early embryogenesis through to adulthood. *Cell Tissue Res.* 292 311–323. 10.1007/s004410051062 9560474

[B135] Takahashi K. Naito M. Katabuchi H. Higashi K. (1991). Development, differentiation, and maturation of macrophages in the chorionic villi of mouse placenta with special reference to the origin of Hofbauer cells. *J. Leukoc. Biol.* 50 57–68. 10.1002/jlb.50.1.57 2056247

[B136] Takahashi N. Udagawa N. Tanaka S. Murakami H. Owan I. Tamura T. (1994). Postmitotic osteoclast precursors are mononuclear cells which express macrophage-associated phenotypes. *Dev. Biol.* 163 212–221. 10.1006/dbio.1994.1137 8174777

[B137] Takasato M. Little M. H. (2015). The origin of the mammalian kidney: implications for recreating the kidney *in vitro*. *Development* 142 1937–1947. 10.1242/dev.104802 26015537

[B138] Tan S. Y. Krasnow M. A. (2016). Developmental origin of lung macrophage diversity. *Development* 143 1318–1327. 10.1242/dev.129122 26952982 PMC4852511

[B139] Thesingh C. W. (1986). Formation sites and distribution of osteoclast progenitor cells during the ontogeny of the mouse. *Dev. Biol.* 117 127–134. 10.1016/0012-1606(86)90355-6 3743892

[B140] Thomas J. R. Appios A. Zhao X. Dutkiewicz R. Donde M. Lee C. Y. C. (2021). Phenotypic and functional characterization of first-trimester human placental macrophages, Hofbauer cells. *J. Exp. Med.* 218:, e20200891. 10.1084/jem.20200891 33075123 PMC7579740

[B141] Tober J. Koniski A. McGrath K. E. Vemishetti R. Emerson R. de Mesy-Bentley K. K. (2007). The megakaryocyte lineage originates from hemangioblast precursors and is an integral component both of primitive and of definitive hematopoiesis. *Blood* 109 1433–1441. 10.1182/blood-2006-06-031898 17062726 PMC1794060

[B142] Travnickova J. Tran Chau V. Julien E. Mateos-Langerak J. Gonzalez C. Lelievre E. (2015). Primitive macrophages control HSPC mobilization and definitive haematopoiesis. *Nat. Commun.* 6:, 6227. 10.1038/ncomms7227 25686881

[B143] Udagawa N. Takahashi N. Akatsu T. Tanaka H. Sasaki T. Nishihara T. (1990). Origin of osteoclasts: mature monocytes and macrophages are capable of differentiating into osteoclasts under a suitable microenvironment prepared by bone marrow-derived stromal cells. *Proc. Natl. Acad. Sci. U. S. A.* 87 7260–7264. 10.1073/pnas.87.18.7260 2169622 PMC54723

[B144] van de Laar L. Saelens W. De Prijck S. Martens L. Scott C. L. Van Isterdael G. (2016). Yolk sac macrophages, fetal liver, and adult monocytes can colonize an empty niche and develop into functional tissue-resident macrophages. *Immunity* 44 755–768. 10.1016/j.immuni.2016.02.017 26992565

[B145] van Furth R. Cohn Z. A. (1968). The origin and kinetics of mononuclear phagocytes. *J. Exp. Med.* 128 415–435. 10.1084/jem.128.3.415 5666958 PMC2138527

[B146] Wang Q. Stacy T. Binder M. Marin-Padilla M. Sharpe A. H. Speck N. A. (1996a). Disruption of the Cbfa2 gene causes necrosis and hemorrhaging in the central nervous system and blocks definitive hematopoiesis. *Proc. Natl. Acad. Sci. U. S. A.* 93 3444–3449. 10.1073/pnas.93.8.3444 8622955 PMC39628

[B147] Wang Q. Stacy T. Miller J. D. Lewis A. F. Gu T. L. Huang X. (1996b). The CBFbeta subunit is essential for CBFalpha2 (AML1) function in vivo. *Cell* 87 697–708. 10.1016/S0092-8674(00)81389-6 8929538

[B148] Wang Y. Szretter K. J. Vermi W. Gilfillan S. Rossini C. Cella M. (2012). IL-34 is a tissue-restricted ligand of CSF1R required for the development of Langerhans cells and microglia. *Nat. Immunol.* 13 753–760. 10.1038/ni.2360 22729249 PMC3941469

[B149] Waqas S. F. H. Hoang A. C. Lin Y. T. Ampem G. Azegrouz H. Balogh L. (2017). Neuropeptide FF increases M2 activation and self-renewal of adipose tissue macrophages. *J. Clin. Invest.* 127:, 3559. 10.1172/JCI95841 28758905 PMC5669560

[B150] Wiktor-Jedrzejczak W. Bartocci A. Ferrante A. W. Jr. Ahmed-Ansari A. Sell K. W. Pollard J. W. (1990). Total absence of colony-stimulating factor 1 in the macrophage-deficient osteopetrotic (op/op) mouse. *Proc. Natl. Acad. Sci. U. S. A.* 87 4828–4832. 10.1073/pnas.87.12.4828 2191302 PMC54211

[B151] Wittamer V. Bertrand J. Y. (2020). Yolk sac hematopoiesis: does it contribute to the adult hematopoietic system? *Cell Mol. Life Sci.* 77 4081–4091. 10.1007/s00018-020-03527-6 32405721 PMC11104818

[B152] Wu C. Li B. Lu R. Koelle S. J. Yang Y. Jares A. (2014). Clonal tracking of rhesus macaque hematopoiesis highlights a distinct lineage origin for natural killer cells. *Cell Stem Cell* 14 486–499. 10.1016/j.stem.2014.01.020 24702997 PMC3979461

[B153] Yahara Y. Barrientos T. Tang Y. J. Puviindran V. Nadesan P. Zhang H. (2020). Erythromyeloid progenitors give rise to a population of osteoclasts that contribute to bone homeostasis and repair. *Nat. Cell Biol.* 22 49–59. 10.1038/s41556-019-0437-8 31907410 PMC6953622

[B154] Yamane T. (2018). Mouse yolk sac hematopoiesis. *Front. Cell Dev. Biol.* 6:80. 10.3389/fcell.2018.00080 30079337 PMC6062755

[B155] Yona S. Kim K. W. Wolf Y. Mildner A. Varol D. Breker M. (2013). Fate mapping reveals origins and dynamics of monocytes and tissue macrophages under homeostasis. *Immunity* 38 79–91. 10.1016/j.immuni.2012.12.001 23273845 PMC3908543

[B156] Yoshida H. Hayashi S. Kunisada T. Ogawa M. Nishikawa S. Okamura H. (1990). The murine mutation osteopetrosis is in the coding region of the macrophage colony stimulating factor gene. *Nature* 345 442–444. 10.1038/345442a0 2188141

[B157] Yoshimoto M. Montecino-Rodriguez E. Ferkowicz M. J. Porayette P. Shelley W. C. Conway S. J. (2011). Embryonic day 9 yolk sac and intra-embryonic hemogenic endothelium independently generate a B-1 and marginal zone progenitor lacking B-2 potential. *Proc. Natl. Acad. Sci. U. S. A.* 108 1468–1473. 10.1073/pnas.1015841108 21209332 PMC3029764

[B158] Yoshimoto M. Porayette P. Glosson N. L. Conway S. J. Carlesso N. Cardoso A. A. (2012). Autonomous murine T-cell progenitor production in the extra-embryonic yolk sac before HSC emergence. *Blood* 119 5706–5714. 10.1182/blood-2011-12-397489 22431573 PMC3382930

[B159] Yzaguirre A. D. Speck N. A. (2016). Insights into blood cell formation from hemogenic endothelium in lesser-known anatomic sites. *Dev. Dyn.* 245 1011–1028. 10.1002/dvdy.24430 27389484 PMC5801748

[B160] Zovein A. C. Hofmann J. J. Lynch M. French W. J. Turlo K. A. Yang Y. (2008). Fate tracing reveals the endothelial origin of hematopoietic stem cells. *Cell Stem Cell* 3 625–636. 10.1016/j.stem.2008.09.018 19041779 PMC2631552

[B161] Zulu M. Z. Martinez F. O. Gordon S. Gray C. M. (2019). The elusive role of placental macrophages: the Hofbauer cell. *J. Innate. Immun.* 11 447–456. 10.1159/000497416 30970346 PMC6758944

